# Challenges That Health Care Systems Faced During and After the COVID-19 Pandemic

**DOI:** 10.3390/healthcare14172731

**Published:** 2026-08-27

**Authors:** Dimitris Zavras

**Affiliations:** Laboratory for Health Technology Assessment, Department of Public Health Policy, School of Public Health, University of West Attica, 196 Alexandras Avenue, 11521 Athens, Greece; dzavras@uniwa.gr; Tel.: (+30)-2105385978

The coronavirus disease 2019 (COVID-19) pandemic represented a global public health and economic crisis, which evolved into a humanitarian crisis, resulting in devastating fatalities and widespread suffering. The health, economic, and societal impacts of the policy responses were unparalleled, manifesting clearly from its initial stages. In combination with the virus dynamics, the restriction measures put excessive strain on healthcare systems. Such consequences were magnified by multidimensional uncertainty concerning the medical, economic, and societal aspects of the crisis. Thus, this editorial aims to delineate the challenges healthcare systems confronted during and after the COVID-19 pandemic and to present the multifaceted implications of this public health crisis for the healthcare sector.

Healthcare systems were ill-prepared for a crisis of such intensity. Even from the onset of the pandemic, significant weaknesses in healthcare systems worldwide emerged. Nearly all countries experienced sudden surges in patient numbers that overwhelmed the healthcare systems [1].

Given the unprecedented strain the pandemic placed on healthcare system capacities, encompassing physical infrastructure like bed availability and medical equipment, as well as healthcare professionals [2], policy responses from healthcare systems can be broadly classified under three core “S” priorities: space, mobilizing staff, and supplies [3], meaning that hospitals should have the ability to expand ICU capacity by extending to other hospital areas, with appropriate beds and monitors for expansion areas; be able to rapidly increase clinical and nonclinical staffing and models of using noncritical care staff to care for ICU patients; and have plans to ensure availability of necessary medical equipment and medications [4].

Moreover, the pandemic had severe consequences for the mortality and morbidity of healthcare workers (HCWs), with a substantial number of this professional group becoming infected and dying globally. Moreover, the psychological well-being of HCWs was significantly affected, as evidenced by the development of mental health symptoms, such as emotional exhaustion [5].

Throughout the pandemic, essential health services experienced disruptions in nearly all countries. Most service disruptions were partial, characterized by a 5–50% alteration in provision or utilization, while severe or complete disruptions entailed an alteration exceeding 50%. No services remained entirely unaffected; however, emergency services experienced the least disruption. Mobile services constituted the most severely impacted service delivery platform. The disruptions were primarily attributable to a combination of demand and supply factors. From the demand perspective, reductions in outpatient care attendance were reported by the majority of countries (76%), largely due to lockdowns impeding access and financial hardships experienced during these periods. Regarding supply, the cancelation of elective services was the most frequently cited factor (66%). Additional factors included the redeployment of staff to support COVID-19 response efforts, the unavailability of services due to the closure of health facilities or services, and challenges within supply chains. National responses predominantly involved the triage of health services, the implementation of telemedicine to substitute for in-person consultations, and modifications to medicine dispensing practices [6]. As the healthcare disruption caused by COVID-19 impacted health service coverage, progress towards achieving universal health coverage (UHC) was consequently undermined [7], while unmet healthcare needs could exert detrimental effects on individuals’ health in the aftermath of such disruptions [8].

Moreover, hospitals and healthcare facilities encountered catastrophic financial challenges stemming from the pandemic. Collectively, insufficient preparedness significantly contributed to the difficulties faced by healthcare facilities globally. Supplies for infection prevention and control (IPC) were notably scarce. Consequently, healthcare organizations globally were compelled to devise new, essential plans for pandemic preparedness [9]. Nevertheless, the COVID-19 pandemic not only exacerbated morbidity and mortality, accompanied by significant economic ramifications for healthcare systems and communities globally [10], but also eroded health equity. This erosion occurred as the pandemic disproportionately impacted impoverished, minority, and various vulnerable populations due to: (i) its uneven propagation in densely populated areas with limited mitigation capabilities, and (ii) the high prevalence of chronic conditions or inadequate access to quality public health and medical care [11].

Since 5 May 2023, COVID-19 does not constitute a public health emergency of international concern.

Broadly, the diminished pressure on global healthcare systems following the world’s emergence from the pandemic can be attributed to (i) the continuing reinforcement after 2020, (ii) the evolution of sophisticated clinical management protocols, and (iii) immunity acquired through infection and/or vaccination [12].

The pandemic acted as a catalyst for change, underscoring the urgent need to implement and adopt reforms in public health interventions. It also pointed to the necessity of a new healthcare delivery model that prioritizes preventive measures, remote care, and substantial technological integration. Nevertheless, these advancements are juxtaposed against persistent technical challenges in meeting surge capacity for laboratory testing, the expedited implementation of new technologies, mental health considerations, ethical dilemmas concerning the potential rationing of scarce resources, and the safeguarding of privacy and personal data during periods of crisis [13].

Regarding healthcare systems, key recommendations primarily encompass investment in health infrastructure, bolstering the healthcare workforce, enhancing access to healthcare, harnessing technology and innovation for system preparedness and response, and fostering collaboration and partnerships [14].

The lessons gleaned from the COVID-19 pandemic have the potential to inform the transformation of global health architecture in anticipation of future outbreaks and global health threats [15].

The pivotal lessons derived from the COVID-19 pandemic can be classified into four domains: (i) the availability of physical resources and a well-trained human workforce, (ii) harmonized and interoperable data and information coupled with accelerated research and development support, (iii) proactive and credible political leadership alongside multisectoral cooperation and coordination, and (iv) transparent and strategic crisis communication ensuring civil society engagement in decision-making processes [16].

Consequently, the three “S” priorities proved instrumental for the recovery of healthcare systems. The impetus for a return to normalcy is driven by this understanding, given the enduring impact of the pandemic on healthcare systems. Over six years since the onset of the crisis, the preparedness of healthcare systems, facilitated by enabling interventions, has emerged as a paramount concern. Nevertheless, from a global perspective, it is imperative to identify the constituent elements of inadequately prepared healthcare systems that encountered significant challenges. This task is readily achievable. The healthcare sector is inherently labor-intensive, a reality that became strikingly clear during the pandemic. Conversely, the existing infrastructure proved inadequate. Therefore, the synergistic effect of vaccination, coupled with the reinforcement of these components and the advancement of clinical management methodologies, facilitated the exit from the pandemic.

Analogous to previous public health emergencies, the recovery from the COVID-19 pandemic signals a need for revised approaches to prevent a recurrence of such a tragedy. Moreover, there must be a renewed acknowledgment of the broader responsibility of healthcare systems to deliver quality and equitable services during routine periods [17]. A consensus regarding the imperative to build resilient health systems has crystallized in the wake of the COVID-19 pandemic. This objective is attainable through five interrelated components: (i) robust financing arrangements; (ii) effective governance structures; (iii) adequate health system resources, public health provisions, and service delivery mechanisms; (iv) comprehensive data and information systems; and (v) a supportive political and socioeconomic context [18].

However, without knowledge of the causes of a catastrophe, it is difficult to address it. Thus, a question is posed: what was the reason for the extent of the influence of COVID-19 on healthcare systems? To answer such a question, we should consider that healthcare systems are complex adaptive systems made up of multiple interacting components. They are open systems that exist in a dynamic relationship with their wider context, i.e., social, political, and economic [19]. The pandemic was also a complex systems problem, as it involved a very large number of diverse and dynamically interacting elements; thus, we should take into consideration the attributes of the crisis, i.e., uncertainty, non-linearity, wide diversity, emergence, unexpected variability, path dependence, self-organization, and resilience [20,21]. All the aforementioned attributes acted as a catalyst for the performance of the healthcare systems during and after the pandemic. The uncertainty was massive, the dynamics of the virus were unpredictable, the effectiveness of the mitigation policies was questioned, and, under review, the social influence was vast even though society attempted to adapt, and resilience was also questioned. Combining such attributes and taking into account that complex systems have memory [22], we may argue that the performance of healthcare systems after the pandemic is subject to the crisis, aiming to foster preparedness and resilience. Although preparedness and resilience are not recent targets of healthcare systems, the pandemic made them imperative. In addition, SARS-CoV-2 remains a global threat for morbidity and mortality.

Using the Biomatrix Tool, the results that healthcare systems want to achieve, in parallel with their unique expectations and values, their structure, the activities involved in the delivery of services to users, the resources, the local and surrounding facilitators and barriers, and the regulation and monitoring, can contribute to their transformation [23]. Based on the aforementioned systemic order, the interactions become more manageable.

The pandemic tested healthcare systems for a prolonged period, pushing them to their limits. Although they have, to a large extent, recovered, the lessons learned from this unprecedented crisis must be accompanied by interventions aimed at enhancing their preparedness and resilience. This will ensure they can adequately cope with similar crises in the future, thereby mitigating their tragic consequences.
